# Characterization of Radiation Shielding Capabilities of High Concentration PLA-W Composite for 3D Printing of Radiation Therapy Collimators

**DOI:** 10.3390/polym16060769

**Published:** 2024-03-11

**Authors:** José Velásquez, Melani Fuentealba, Mauricio Santibáñez

**Affiliations:** 1 Departamento de Ciencias Físicas, Universidad de La Frontera, Temuco 4811230, Chile; 2 Laboratorio de Radiaciones Ionizantes, Universidad de La Frontera, Temuco 4811230, Chile; 3 Programa de Doctorado en Ingeniería, Facultad de Ingeniería y Ciencias, Universidad de La Frontera, Temuco 4811230, Chile

**Keywords:** PLA-W, SFGRT, PLA-metal composites, grid therapy

## Abstract

This work evaluates the radiation shielding capabilities of the PLA-W composite for MV energy photons emitted by a linear accelerator and the feasibility of manufacturing a clinically-used collimator grid in spatially fractionated radiotherapy (SFRT) using the material extrusion (MEX) 3D printing technique. The PLA-W filament used has a W concentration of 93% *w/w* and a green density of 7.51 g/cm^3^, characteristics that make it suitable for this purpose. Relevant parameters such as the density and homogeneity distribution of W in the manufactured samples determine the mass attenuation coefficient, directly affecting the radiation shielding capacities, so different printing parameters were evaluated, such as layer height, deposition speed, nozzle temperature, and infill, to improve the protection performance of the samples. Additionally, physical and mechanical tests were conducted to ensure structural stability and spatial variability over time, which are critical to ensure precise spatial modulation of radiation. Finally, a complete collimator grid measuring 9.3 × 9.3 × 7.1 cm^3^ (consisting of 39 conical collimators with a diameter of 0.92 cm and center-to-center spacing of 1.42 cm) was manufactured and experimentally evaluated on a clinical linear accelerator to measure the radiation shielding and dosimetric parameters such as mass attenuation coefficient, half-value layer (HVL), dosimetric collimator field size, and inter-collimator transmission using radiochromic films and 2D diode array detectors, obtaining values of 0.04692 cm^2^/g, 2.138 cm, 1.40 cm, and 15.6%, respectively, for the parameters in the study. This shows the viability of constructing a clinically-used collimator grid through 3D printing.

## 1. Introduction

3D printing has undergone prompt development due to its accessibility and usefulness in various fields of science and engineering, and it has been rapidly integrated into various biomedical fields [1,2,3,4]. Its operation is based on additive manufacturing (AM), which is part of a set of techniques in which three-dimensional structures are built layer by layer by an automated mechanism [5,6,7]. This method offers several advantages over traditional subtractive or conformal manufacturing techniques, such as the production of more complex geometries, the use of multiple materials in the same part, the elimination of tooling, and lower production costs [7,8]. Among the various options for AM techniques, the material extrusion process (MEX), such as fused filament fabrication (FFF), is one of the most widely used [9,10]. This modality uses a continuous filament of thermoplastic material, most commonly polylactic acid (PLA) and Acrylonitrile Butadiene Styrene (ABS) [11,12,13].

Polymer 3D printing has multiple applications in the clinical area, and one that has aroused great interest is its radiation shielding capabilities [14]. Several works have experimentally evaluated the very low energy radiation shielding capacity of various commercially available polymers [15,16]. However, the development of metal oxide PLA composites has allowed for a greater advantage in this area [17,18]. Computer simulation work using the Geant4 Monte Carlo code has evaluated the potential capacity that PLA compounds doped with oxides of Mg, Fe, Zn, and Ti [17] and polyimide compounds doped with Zn, Ti, Ag, and Au [19] would have for photon and neutrons shielding in a wide energy range (10–2000 keV). Additionally, polymers doped with Ag nanoparticles have also been experimentally analyzed for their effectiveness in shielding against 662 keV gamma rays [20]. Recently, the experimental evaluation of PLA doped with low concentrations of Pb [18] showed the feasibility of increasing the radiation shielding capacity for photons of energies in the keV range. Additionally, W has been studied for its effectiveness as a radiation shield in electronic devices [21] and superficial electron radiotherapy [22].

However, the deposition of PLA-metal composites has several challenges that critically determine the radiation shielding capacity, including the printing parameters such as the extrusion temperature, the deposition speed of the PLA-metal composite, the layer height, the infill, and the metal concentration in the PLA composite [23]. These parameters determine the distribution of the metal in the deposited layer, generating alterations in the density and homogeneity of the material. The former variable dictates the material’s linear attenuation capacity [24], while the latter governs dosimetric spatial inhomogeneity [25]. Although microscopic heterogeneities are always expected, they result in dosimetric discrepancies smaller than the uncertainties inherent in dosimetric measurements obtained through current international protocols [26,27]. Therefore, the evaluation of the quality of its shielding behavior and the achieved dosimetry will be analyzed exclusively based on the macroscopic averages of these variables.

To date, most existing applications have evaluated the radiation attenuation capacity for photon energies primarily in the keV range [18,20], neglecting an important area of clinical application such as MV photon radiotherapy. Historically, radiotherapy has required the use of compensators and collimators [28], which enable the transmission of radiation to be defined and confined to a spatial region, minimizing the contribution to external regions as much as possible, thus reducing incident radiation to levels typically ranging from 15% to 30% of the maximum peak [29]. Modern radiotherapy uses electron linear accelerators (6–18 MV), which produce photons with MeV energies, with the capacity to penetrate matter significantly more than the keV energies assessed thus far [24]. For a medium such as PLA or Polyethylene, the linear attenuation coefficient for the 20–100 keV range is 0.7773–0.1996 cm^−1^ [19,24] whereas for the same materials at energies in the 1–6 MeV range the linear attenuation coefficients are in average 8.4 times lower (0.0838–0.0324 cm^−1^) [19,24], which will result in an attenuation 3–4 orders of magnitude lower for 1–6 MeV photons. This suggests that the production of any type of radiotherapy accessory by MEX would require polymeric compounds doped with high Z and high-density metals (such as W or Pb with linear attenuation coefficients of 1.2740–0.8104 cm^−1^ and 0.8054–0.4979 cm^−1^ respectively for 1–6 MeV [24]) in concentrations exceeding 90% to achieve attenuation of the radiation beam to 1/7 of its value in reasonable thickness dimensions. In recent years, commercial brands have introduced 3D filaments with high concentrations of W (91% to 93% *w*/*w*), boasting a green density of 7.5–8.0 g/cm^3^ [30], opening new opportunities.

An interesting application in the field of MV radiotherapy involves the creation of multi-collimator blocks for spatially fractionated radiotherapy (SFRT). SFRT is a treatment method that involves administering high doses of radiation in a single fraction to a target volume in a heterogeneous manner using individual thin beams [31]. This approach utilizes accessories like grid-shaped blocks made with high-density materials, offering advantages for specific pathologies not achievable with modern techniques [31,32,33]. These grid collimators were traditionally constructed using CNC machining techniques outside hospital environments or purchased commercially [34] at a high cost as generic grids without customization by pathology or patient. Alternatively, in-hospital manufacturing has been reported using Cerrobend alloys (consisting of Pb, Bi, Sn, and Cd) [35]; however, due to the highly toxic Cerrobend components, its use has been progressively eliminated in clinical centers [36]. Over the last few years, 3D printing has emerged as an alternative for manufacturing grid collimators by creating a negative mold from creep maps, into which a liquid-based Cerrobend compound is poured to obtain a solid compensator [35], or by using tungsten balls [37].

When directly 3D printing the grid, precise alignment of the manufactured parts becomes crucial, especially for precision applications requiring specific attenuation or transmission tolerances. However, many polymeric materials commonly used in 3D printing have lower mechanical strength compared to those produced through reinforced injection molding techniques [38,39]. Even the case of PLA compounds doped with metals (e.g., Fe, Cu, Bronze, and Stainless Steel) has been widely reported, where in an attempt to enhance other physical properties of the material, they have considerably reduced their tensile strength, resulting in mechanical properties inferior to pure PLA [40]. Quality analysis and functionality testing of an accessory manufactured by 3D printing with filaments doped with high Z elements, used as a radiation-blocking collimator, mainly involve checking for deformations during insertion into the electron linear accelerator and its movement in the equipment head rotation [41].

The aim of this study is to determine the feasibility of manufacturing SFRT treatment grids using the MEX 3D printing technique with PLA-W, achieving clinical standards of dosimetric accuracy. This involves achieving specific levels of shielding and homogeneity in the manufactured material, as well as ensuring precision and mechanical stability during its use. Our approach involves maximizing radiation attenuation and homogeneity by varying printing parameters. Additionally, we assess whether the potential loss of mechanical properties in PLA-W could lead to grid deformations, thereby altering its dosimetry. Dosimetric characterization adhered to strict international protocols, including IAEA TRS-457 [42], IAEA TECDOC1151 [41], IAEA TRS-398 [26], and AAPM TG-51 [27]. These protocols facilitated the measurement of attenuation and spatial homogeneity, as well as the characterization of shielding properties via the determination of HVL and spatial dose delivery by the grid across various regions of interest.

## 2. Materials and Methods

### 2.1. Material and Printing Properties

#### 2.1.1. Material Composite

**Figure 1:**
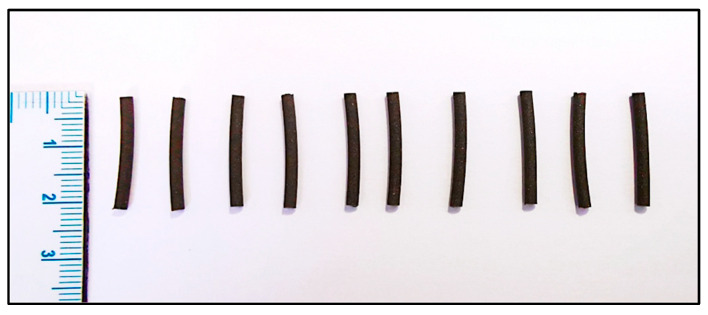
Samples of 10 pieces of filament of 2 cm length were used for filament density measurement.

The selected material is a PLA polymeric composite with tungsten (PLA-W), specifically the Rapid 3D Shield model from The Virtual Foundry brand (The Virtual Foundry, Inc. Stoughton, WI, USA) [30]. These filaments have a variable composition of compounds specified in each spool. The filament used consists of 6.9% PLA and 93.1% W powder, with a density of 7.51 g/cm^3^ (material details in CAS 7440-33-7) [43] and a filament diameter of 1.75 ± 0.05 mm. For quality control purposes, the diameter and density of the spool filament were evaluated. The filament density was determined according to ASTM D792 [44] by analyzing 10 random samples, each 2 cm in length (see Figure 1). The diameter of these samples was measured using a vernier caliper with a resolution of 0.01 mm.

#### 2.1.2. 3D Printer Device

The 3D printer used was the PRUSA i3 MK3S model (Prusa Research a.s., Prague, Czech Republic), which is recommended for this type of filament since it contains metallic particles and is more fragile than conventional filaments. The Prusa i3 MK3S model has a direct extrusion feeding mechanism. In MEX, the retraction movements of the filament are used to avoid excess material due to internal/external pressure differences between displacements of the printer nozzle. If this functionality is not activated, imperfections may appear in the manufactured piece, typically as drops on the surface and on threads. This issue is accentuated if the filament diameter is not constant, which is relevant in the case of the W-PLA compound since it is susceptible to deformations caused by the extrusion rollers. For this reason, a printer that operates with Bowden-style retractions experiences greater elongation in the filament, resulting in a loss of regularity of the expected diameter. This system could damage the printing process, as the extruder is located in the printer frame and reaches the hot end through a polytetrafluoroethylene tube rather than being mounted directly on the printhead [45,46].

According to the manufacturer’s description, this composite can be manufactured using a 0.6 mm diameter hardened steel nozzle, owing to the high level of abrasion caused by the metallic particles with which the PLA polymer is doped. Moreover, smaller-sized printing nozzles, starting from 0.4 mm and below, are more likely to become occluded with the W filament due to higher pressure [6,45], potentially leading to irregularities in the exit diameter and affecting sample resolution.

#### 2.1.3. Printing Settings Parameters

**Figure 2:**
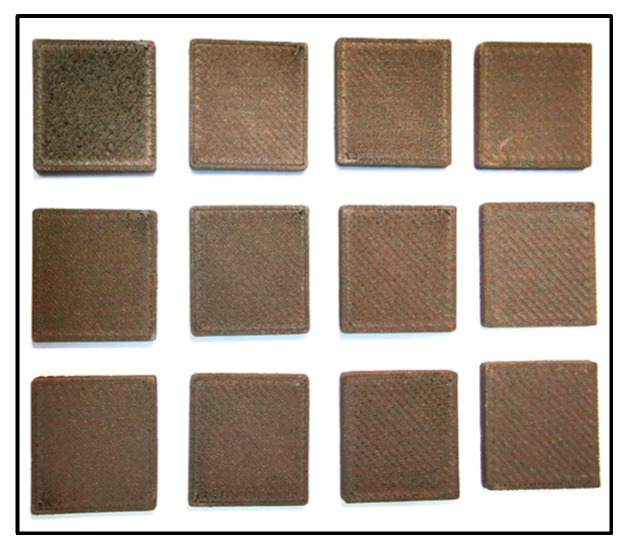
Samples from the 12 PLA-W slabs of dimensions 20 × 20 × 1.8 mm^3^.

To enhance the attenuation and radiation shielding homogeneity properties of the PLA-W manufactured pieces, the dependence of these properties was analyzed with respect to printing parameters. These parameters included layer height (0.15, 0.30, and 0.45 mm), deposition speed of 75%, 100%, and 150% of a nominal 70 mm/s, and nozzle temperature ranging from 205 °C to 235 °C. For comparison purposes, 12 slabs of the material, each measuring 2.0 × 2.0 cm^2^, were fabricated using the parameters described above. Detailed parameter settings are provided in Table 1 and graphically illustrated in Figure 2.

### 2.2. Physical and Mechanical Properties Analyzed

The primary objective of the various physical and mechanical tests conducted was to analyze and predict potential macroscopic discrepancies between the geometry of the manufactured pieces and the designed ones. These discrepancies may arise from structural alterations over time or from stresses encountered during their application, directly impacting radiation shielding and the corresponding delivered dosimetry.

For each sample, verification of the physical dimensions acquired during manufacturing (height, length, thickness), the final mass, and the obtained density was conducted. This assessment aimed to evaluate how the printer parameters affect the accuracy of the final sample. Physical dimensions and mass measurements were taken using a vernier caliper (resolution 0.01 mm) (Mitutoyo, Sakado, Kawasaki, Japan) and an analytical balance (resolution 0.1 mg) (Quimis Scientific Instruments, Diadema, SP, Brasil). Additionally, the density of all manufactured samples was assessed according to ASTM D792 standards [44]. This information helps determine the mass thickness of attenuation obtained for each sample, which is necessary for subsequent standardized radiation attenuation tests such as IAEA TECDOC 1151 [41].

**Figure 3:**
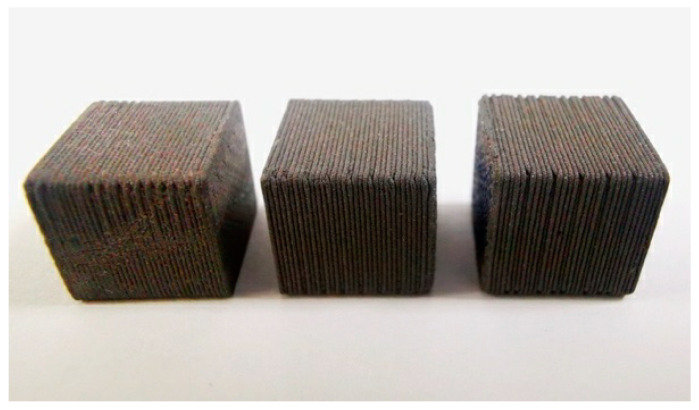
1.0 × 1.0 × 1.0 cm^3^ cubes of PLA-W in ascending order of infill percentage: 15%, 50%, and 100% (from left to right).

The infill percentage parameter is another variable that directly influences the density of the print. To investigate this, three cubes measuring 1.0 × 1.0 × 1.0 cm^3^ were designed and printed, as depicted in Figure 3. The printing process was conducted using a 0.6 mm diameter nozzle and three different infill percentages: 15% (within the standard range for maintaining the structural properties of the piece) [47,48], 50%, and 100%. The cube density was also determined according to the ASTM D792 norm [44].

To evaluate material variability over time (with the aim of detecting alterations in the collimators that may occur throughout a course of radiotherapy treatment.), the cube dimensions were measured with a vernier caliper (resolution 0.01 mm) and repeated 12 months later after the cubes had been stored under constant temperature and humidity conditions of 22 °C and 65%. These conditions represent the average environmental conditions of the facilities where the accessories exposed to radiation are stored.

Likewise, mechanical tests were conducted with the aim of analyzing whether the anticipated decrease in the mechanical properties of the PLA-W could cause modifications in the geometry of the collimators due to the forces generated by the weight of the collimator itself or by the displacements resulting from the different angular positions in the clinical equipment where they are used. This could alter the spatial modulation patterns of radiation.

**Figure 4:**
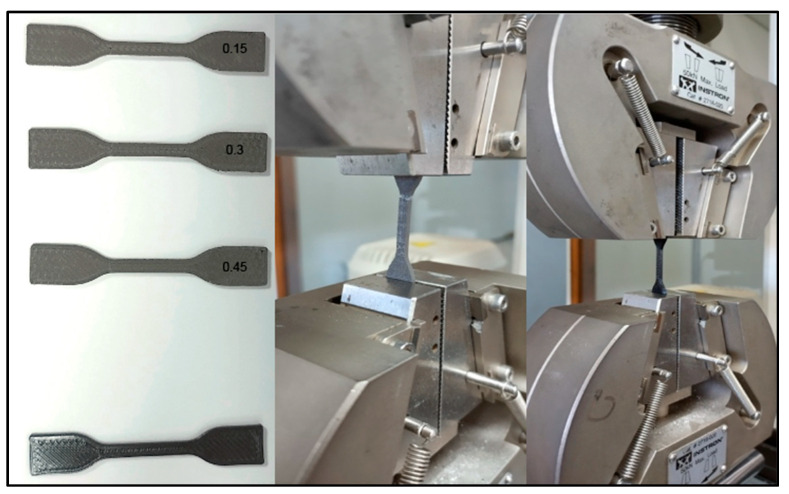
PLA-W specimens with a variable layer thickness (0.15, 0.30, and 0.45 mm) and PLA specimen samples for tensile evaluation (**left**). PLA-W and PLA in mechanical tensile stress evaluation (**center** and **right**).

The mechanical characterization was conducted following the ASTM D638 type IV standards [49], which involved preparing specimens of both the metallic composite under evaluation and the reference material (PLA). The specimens were designed on the adjustment parameters of the MEX process outlined in Table 2. The test specifically evaluated tensile stress, as illustrated in Figure 4.

### 2.3. Radiation Properties

#### 2.3.1. Radiation Shielding Homogeneity

**Figure 5:**
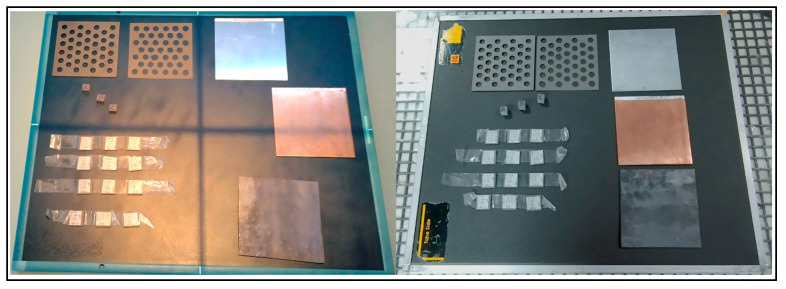
Samples of 12 slabs prepared with variation of printing parameters distributed in the order indicated in Table 1 (lower left corner), together with copper, aluminum, and lead filters (right) arranged in digital chassis for evaluation of kilovoltage radiography (left image) and 6 MV scintigraphy (right image).

The evaluation of the attenuation and radiation shielding homogeneity achieved for each printing setting was obtained by means of X-ray digital images of kV and MV energies. The kV images were acquired using Philips model DigitalDiagnost C50 diagnostic radiography equipment (Philips Healthcare, Amsterdam, The Netherlands) with acquisition parameters of 150 kVp, 100 mA, and 50.0 ms. These images were processed using an AGFA Healthcare model DX-M CR (computed radiography) system (Agfa-Gevaert Group, Mortsel, Belgium), which facilitates image digitization while preserving high spatial resolution. Similarly, MV images were obtained with a Varian model UNIQUE 6 MV clinical linear accelerator (Varian Medical Systems, Palo Alto, CA, USA) in a digital chassis (refer to Figure 5).

The geometry of the samples manufactured for analysis was established based on the following criteria: thickness was limited according to the achievable quality of digital X-ray images at kV and MV energies. Samples with elevated thickness may produce “image artifacts”, such as distortion or the generation of non-existent images, due to radiation scattering effects within the sample. Along with the PLA-W samples, images of three GAMMEX RMI X-ray filters (GAMMEX, Middleton, WI, USA) made of materials with different densities, Al, Cu, and Pb, each measuring 2.0 mm in thickness, were captured to create an attenuation calibration curve based on pixel intensity within the estimated PLA-W range, following the international IAEA TRS-457 code of practice [42]. In order to generate digital images within the same contrast range for image processing, a sample thickness was chosen to be as similar as possible to the filter thickness and achievable with the different layer height configurations studied (1.85 mm thickness). Finally, the filters have lateral dimensions of 2.0 cm, as the typical inter-collimator spacing of commercially available grids ranges from 1.2 to 2.0 cm [50]. Thus, it is of interest to assess the radiation shielding homogeneity on a surface of similar dimensions. 

The images were analyzed using ImageJ image processing software (Version 1.54). The DICOM format images were transformed to 8-bit grayscale using the National Institutes of Health (NIH) grayscale standard [51]. Utilizing the calibrated images, regions of interest (ROI) of 15 × 15 mm^2^ were defined for each of the slabs to evaluate the mean transmittance value of the radiation and scattering along the ROI.

#### 2.3.2. Material Attenuation

Since the main objective of this study is to evaluate the radiation attenuation capability of the PLA-W composite, a theoretical estimation of the mass attenuation coefficient μ_m_ in units of cm^2^/g was performed for the energy range of 1–6 MeV and an experimental evaluation for clinical accelerator energy of 6 MV.

In the case of a compound of two or more elements, the mass attenuation coefficient is obtained from Equation (1):

(1) $${\left(\mu_{m}\right)}_{comp}=\sum w_{i}{\left(\mu_{m}\right)}_{i}$$

where $w_{i}$ is the mass concentration of the i-th element in the sample and ${\left(\mu_{m}\right)}_{i}$ is the attenuation coefficient of each element in the compound. For the case of the PLA-W composite studied, the mass concentration of each element is shown in Table 3.

The attenuation coefficients for each element within the 1–6 MeV energy range were sourced from the National Institute of Standards and Technology (NIST) XCOM database [24].

For experimental determination, the Lambert-Beer law, as expressed in Equation (2), was utilized to measure the material’s attenuation:

(2) $$I=I_{0}e^{-\left(\mu_{m}\right)\rho x}$$

where *I* correspond to the radiation intensity recorded by a suitable detector that is transmitted through the attenuating material, $I_{0}$ is the radiation intensity recorded by the same detector in the absence of the attenuating material, $\left(\mu_{m}\right)$ is the mass attenuation coefficient of the material, ρ the density of the material and x is the physical thickness of the material. A characteristic parameter of the Lambert-Beer law is the so-called half-value layer (HVL), which corresponds to the physical thickness of material that can halve the transmitted radiation intensity, which is obtained from Equation (3):

(3) $$HVL=\frac{ln\;2\;}{\left(\mu_{m}\right)\cdot \rho }\;\to \;\left(\mu_{m}\right)=\frac{ln\;2\;}{\rho \cdot HVL}$$

**Figure 6:**
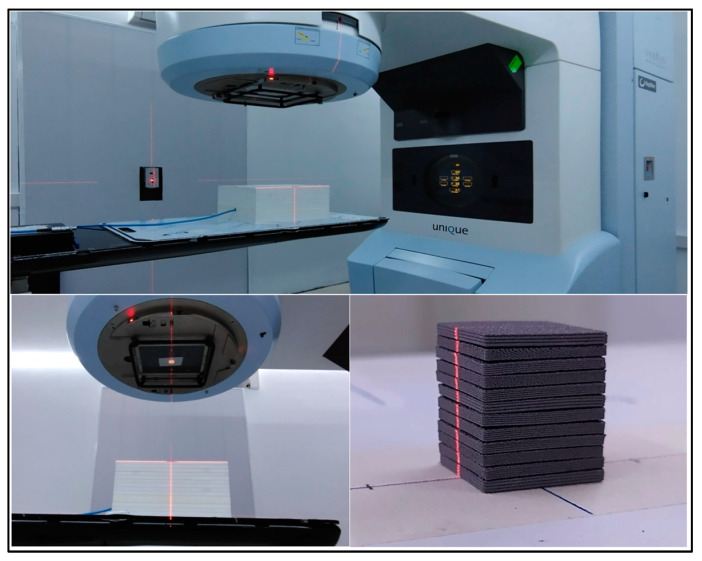
Experimental setup for HVL determination with a UNIQUE linear accelerator and PLA-W slabs in solid water phantom with an ionization chamber (**top**). Enlarged image of setup with 3.6 × 3.6 cm^2^ irradiation field (**left**). PLA-W filter distribution (**right**).

The HVL determination of the PLA-W polymeric compound for the 6 MV UNIQUE clinical accelerator involved assessing the decrease in dose following IAEA TECDOC 1151 quality control protocol [41], measured by a PTW Semiflex 31013 cylindrical ionization chamber (PTW-Freiburg, Baden-Württemberg, Germany). This was achieved by inserting successive slabs of 1.68–1.82 mm thickness (equivalent to mass thicknesses of 1.159–1.274 g/cm^2^). Dose measurement was carried out according to international dosimetric protocols, with the ionization chamber positioned inside a 30 × 30 cm^2^ Sun Nuclear Solid Water equivalent plastic slabs (Sun Nuclear Corporation, Melbourne, FL, USA). The setup included a depth of 10 cm, 5 cm of equivalent Solid Water on the backside for backscatter contribution, a surface source distance (SSD) of 100 cm, and a square field size of 3.6 × 3.6 cm^2^. Square sheets measuring 2.0 × 2.0 cm^2^ and 1.8 mm thick, manufactured with PLA-W using the aforementioned enhancement parameters, were placed 46.5 cm from the slab surface to establish an attenuation curve ranging from 0 to 2.104 cm (0–14.533 g/cm^2^) as shown in Figure 6. The first HVL value was determined by fitting a descending exponential curve of the normalized ionization reading data against mass thickness (g/cm^2^), with the fitted exponential coefficient representing the mass attenuation coefficient. This data additionally facilitated the determination of the effective energy of the radiation spectrum emitted by the accelerator.

### 2.4. Design and Characterization of the Radiotherapy Collimator Grid

**Figure 7:**
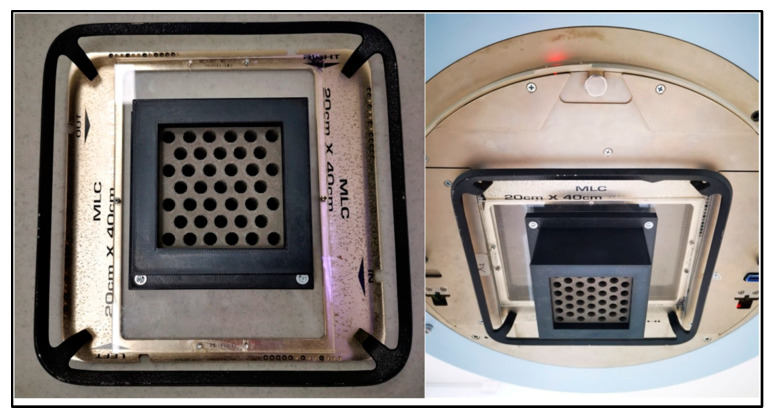
(**Left**) A grid made of PLA-W with honeycomb pattern holes mounted on a UNIQUE linear accelerator tray and (**Right**) grid inserted in linear accelerator head (seen from below).

Currently, grid designs are reported to optimize the passage of the radiation beam while respecting its divergence. The construction of this grid was based on the design published by A. Nobah [52], corresponding to a block of 9.3 × 9.3 × 7.1 cm^3^ (Figure 7). The grid consists of 39 divergent conical holes arranged in a hexagonal honeycomb pattern, each with an exit diameter of 0.92 cm and a center-to-center separation of 1.42 cm. The grid size allows projecting a maximum irradiation field size of 14 × 14 cm^2^ with the isocenter located at a distance of 35.1 cm from the grid edge (the distance from the irradiation source to the plane is 100 cm). The theoretical projection of the grid at the isocenter indicates a hole size of 1.42 cm and a separation between its centers of 2.19 cm.

#### 2.4.1. Field Size and Beam Divergence

The evaluation of the actual dosimetric field size generated by the manufactured collimators and the inter-collimator separation, both at the exit of the grid and in the projection to the isocenter, was obtained by irradiating Ashland model EBT3 radiochromic films (Ashland Advanced Materials, Bridgewater, NJ, USA). The EBT3 films were placed in two positions: the first one was located immediately at the exit of the central hexagon of the grid collimator (formed by seven collimators), and the second one was located at the isocenter. The films were digitized using a dedicated EPSON model Perfection V850 PRO transmission flatbed scanner (Seiko Epson Corporation, Suwa, Nagano, Japan) and processed using ImageJ software version 1.43. During digitization, the image was simultaneously acquired from a sub-millimeter scale microscope ruler to calibrate the pixel dimensions to physical dimensions. The evaluation of the films to be analyzed was performed using a calibration curve created by irradiating films at known doses under reference conditions according to the IAEA TRS-398 high-energy X-ray protocol [26], which considers a field size of 10 × 10 cm^2^, a SSD of 100 cm, and a mass depth of 10 g/cm^2^. The beam divergence generated by the collimators was also evaluated by two methods: the first using the inverse square law of the distance from the dimensions of the dosimetric field measured at the exit of the collimator and the isocenter, and the second by recording the beam propagation on EBT3 films placed perpendicular to the grid at the isocenter, forming a sandwich configuration with a set of plastic slabs of water-equivalent material to maintain their alignment and position respective to the beam. The irradiated films were digitized, as previously mentioned earlier.

#### 2.4.2. Radiation Shielding Capability

To evaluate the radiation shielding capability of the manufactured collimator grid, the relative dose distribution was determined in a plane perpendicular to the radiation beam located at the isocenter, produced by irradiation with the grid installed at the exit of the Varian UNIQUE linear accelerator. Dose distributions were evaluated using two detector systems: the first with a Sun Nuclear MapCheck solid state (diodes) detector array (Sun Nuclear Corporation, Melbourne, FL, USA) calibrated following the protocol recommended by the manufacturer and irradiating the matrix with a known dose obtained from absolute dosimetry performed on the accelerator according to the IAEA TRS-398 protocol for a field of 10 × 10 cm^2^ and the output factor obtained for a field of 20 × 20 cm^2^ [26]. For the test, the diode matrix was located at the isocenter at an effective water-equivalent depth of 2 cm, irradiated with a field size of 10 × 10 cm^2^ and a prescribed dose of 1 Gy. The recorded data are visualized via isodose curves of the regions defined by the collimator holes and the inter-collimator regions to evaluate the transmittance in the blocking zones. The second method considered EBT3 films located at the isocenter at a depth of 5 cm in water-equivalent plastic slabs and irradiated with a field of 10 × 10 cm^2^ and a prescribed dose of 5.0 Gy. To obtain relative numerical values of the radiation attenuation of the inter-collimator regions concerning their centers, the pixel intensities or optical densities of the different regions of the EBT3 film images digitized by the transmittance scanner need to be transformed to doses through a calibration curve. The dose-response calibration was implemented for a range of 0.5–5.0 Gy by digitizing the images of the films irradiated with each of the doses in RGB mode at 16 bits per channel, 96 dpi, and using the red channel for all the analyses.

## 3. Results

### 3.1. Material and Printing Properties

#### 3.1.1. Material Composite

The analysis of the density of the filament under study, performed on a sample of 10 specimens, yielded an average density of 7.89 ± 0.04 g/cm^3^. Although this falls within the range of densities for marketed filaments according to manufacturer-reported data (7.5–8.0 g/cm^3^), it is higher than the density indicated on the spools used (7.51 g/cm^3^). On the other hand, the filament diameter verification fell within the expected range, with an obtained average of 1.75 ± 0.05 mm (reported value 1.75 ± 0.03 mm). The slightly higher uncertainty is explained by the resolution (0.01 mm) and accuracy of the instrument used (0.02 mm).

#### 3.1.2. Physical Properties Analyzed as a Function of Printing Parameters

The evaluation of the influence of printing parameters on spatial congruence and the amount of material deposited on the manufactured samples, which collectively affect the final density achieved, is summarized in Table 4. The results indicate that increasing the deposition speed (ranging from 52.5 to 105 mm/s) for layer heights of 0.15 mm (samples 1, 2, and 3) and 0.30 mm (samples 4, 5, and 6) results in a decrease in achieved density by up to 3.7% and 5.1%, respectively. However, for the largest layer height studied, 0.45 mm (samples 7, 8, and 9), equivalent to 75% of the extruder nozzle diameter, this effect is less significant, with no variations greater than 1%. The effect on the spatial congruence of the samples is observed as a function of the layer height, where for the samples with 0.45 mm layers, a discrepancy in the configured physical dimensions of −0.38% is obtained, while for 0.15 mm and 0.30 mm, the discrepancy reaches −1.38% and −4.83%, respectively. Finally, the nozzle temperature has a directly proportional effect on the density, with a minimum of 6.02 g/cm^3^ for the minimum recommended temperature of 205 °C (sample 10) up to a value of 7.18 g/cm^3^ for 235 °C (sample 12). These densities are considerably lower than those reported by the manufacturer and those obtained experimentally directly from the filament.

The verification of density as a function of the infill percentage of regular cubic structures of 1.000 cm^3^ yielded the following results. When comparing the densities of the cubes with respect to the filament, it is observed that the sample manufactured with a 100% infill contains a lower density than that obtained for the filament on the spool. The measured density was 7.15 ± 0.02 g/cm^3^, which is 9.4% and 4.9% lower than what was measured using the same standard for the filament and what was reported by the manufacturer. At the other extreme, for an infill of only 15% (typically used in 3D printing manufacturing), a density of only 4.60 ± 0.01 g/cm^3^ is achieved.

If the behavior of the final physical dimensions of the manufactured product is evaluated as a function of the infill, it is observed that, for percentages lower than 50%, the discontinuous PLA structures generated in each layer tend to collapse more under the weight exerted by the W material, resulting in smaller dimensions than those designed. In particular, for the samples under study, an infill of 15% resulted in a volume of 0.932 cm^3^ ± 0.006 cm^3^, while an infill of 50% yielded 0.946 cm^3^ ± 0.006 cm^3^, representing a discrepancy of −6.8% and −5.4% respectively, compared to the designed volume of 1.000 cm³. On the other hand, the sample with a 100% infill obtained a volume of 1.027 cm³ ± 0.006 cm³, corresponding to a discrepancy of only +2.7% in relation to the design. Similarly, the evaluation of the stability of the dimensions of the manufactured product over a year showed that no discernible modifications were generated beyond the experimental measurement uncertainty (see Table 5).

### 3.2. Mechanical Properties

**Figure 8:**
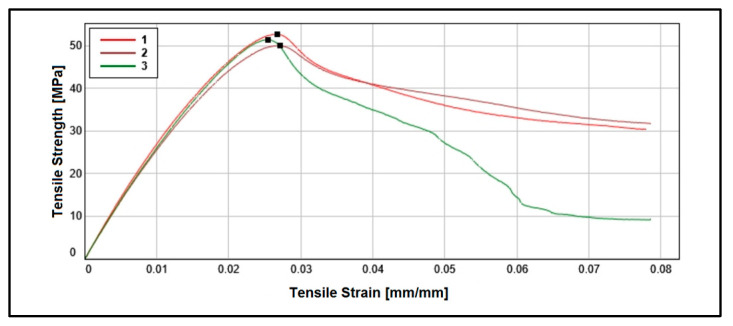

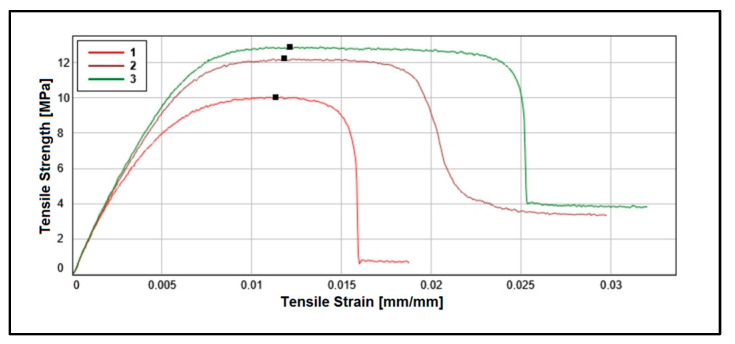
Stress-strain curves of PLA (**above**) and PLA-W (**below**), where curves 1 (red line), 2 (burgundy line), and 3 (green line) correspond to layer heights 0.15, 0.3, and 0.45 mm, respectively.

**Figure 9:**
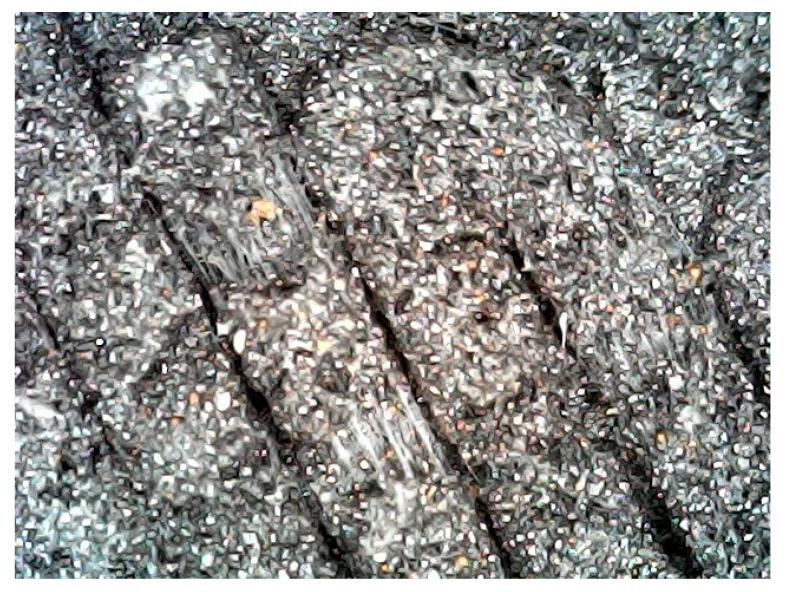
Submillimeter image of the PLA-W manufactured layers showing the discontinuities of the PLA fibers in the structure.

Since the tungsten powder present in PLA-W is not introduced to enhance the mechanical properties of the polymer but rather to improve its radiation shielding properties, a decrease in Young’s modulus and tensile strength values of PLA-W with respect to PLA is observed. Similar results have been reported for other metals such as Fe, Cu, Bronze, and Stainless Steel [40], decreasing the mechanical properties that are directly related to PLA fiber interruptions, as illustrated in Figure 8. The relative decrease of Young’s modulus in PLA-W/PLA showed reductions as the layer height increased, with differences of −20.99%, −16.36%, and −15.67% for layer heights of 0.15, 0.30, and 0.45, respectively. On the other hand, the tensile stress shows a more significant variation, up to −80.91% for a layer height of 0.15, which decreases slightly as the layer height increases with a variation of −74.89% for a layer height of 0.45. The results are summarized in Table 6. Additionally, when observing the complete stress-strain curve, it is evident that an abrupt rupture occurs without an elongation phase for values after the maximum value of tensile stress (see Figure 9), which is relevant data when considering the direct mechanical forces that will act on the manufactured part.

### 3.3. Radiation Properties

#### 3.3.1. Radiation Shielding Homogeneity

The results of the attenuation and radiation shielding homogeneity, assessed through X-ray digital images of kV and MV energies after the 8-bit grayscale calibration process [50], are summarized in Table 7.

The results indicate that all samples exhibit similar homogeneity in macroscopic radiation attenuation and protection, as evidenced by the standard deviation of pixel intensities in the images. Although configurations may experience microscopic inhomogeneities, these effects are deemed insignificant in dosimetric terms for the application under study. Upon analyzing the radiation attenuation capacity, it was observed that the highest values are attained with high deposition velocities (105 mm/s) and maximum layer heights of 0.45 mm. Additionally, the extrusion nozzle temperature parameter proves to be highly influential, with the highest radiation attenuation values achieved at the highest temperatures studied (235 °C).

#### 3.3.2. Material Attenuation

**Figure 10:**
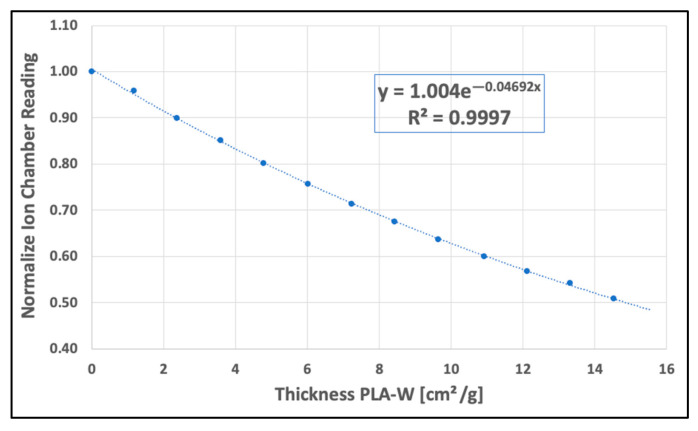
Experimental attenuation with different PLA-W thicknesses.

From the described chemical composition of the material, a mass attenuation coefficient ranging from 6.628 × 10^−2^ cm^2^/g for 1 MeV energy to 3.950 × 10^−2^ cm^2^/g for 4 MeV, was obtained from the NIST database, which subsequently increases monotonically with increasing energy, reaching a value of 4.039 × 10^−2^ cm^2^/g for 6 MeV. Results are shown in Table 8:

On the other hand, the experimental evaluation of the attenuation generated by successive PLA-W layers for a 6 MV clinical accelerator is shown in Figure 10. An exponential fit was achieved according to the expected behavior of the radiation attenuation in the medium with a goodness of fit of R^2^ = 0.9997. From the fitting parameter in the exponent, a mass attenuation coefficient of 0.04692 cm^2^/g was obtained, which, interpolating between the values in Table 8, represents an effective energy of the clinical linear accelerator of 1.747 MeV. The average measured density of the PLA-W slabs used was 6.91 g/cm^3,^ which determines a value of the first HVL of the material of 2.138 cm. These values are summarized in Table 9. 

### 3.4. Design and Characterization of the Radiotherapy Collimator Grid

#### 3.4.1. Field Size and Beam Divergence

**Figure 11:**
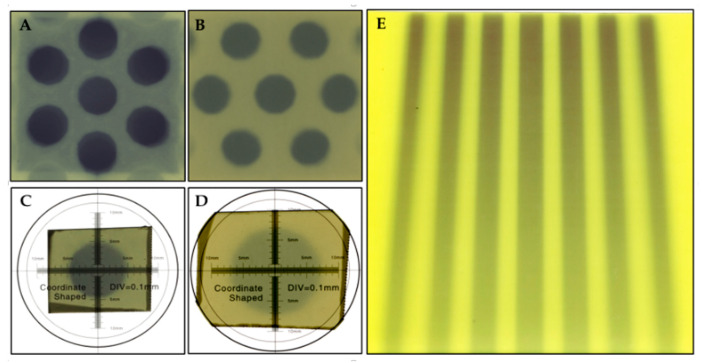
Images of grid measurements with EBT3 radiochromic films. Each image set of the grid is in order: at the grid exit (**A**,**C**) and a distance of 35.1 cm from the grid at the isocenter (**B**,**D**). (**A**,**B**) show the hexagonal array to measure distances while (**C**,**D**) shows the calculation of the divergence of the central hole. On the right (**E**), an image of the radiation fields on the central axis of the holes with their respective divergences at the isocenter with a field aperture of 13 × 13 cm^2^.

The recording of the radiation field produced on the EBT3 films located at the exit of the collimating grid and the isocenter for the hexagonal array of central collimators is shown in Figure 11A,B. The radiation diameters of the central grating aperture measured by the spatial calibration performed with the microscope ruler (Figure 11C,D) yielded a value of 0.91 ± 0.01 cm at the lower edge of the grid and 1.40 ± 0.01 cm at the isocenter, which differed by less than 1.1% and 1.4% of the designed values, respectively. Similarly, the evaluation of the distance between the collimators yielded a result of 1.40 ± 0.01 cm at the exit of the collimators and 2.27 ± 0.01 cm at the isocenter, which differed by less than 1.4% and 3.6%, respectively. The discrepancies are in the sub-millimeter range, which, in the case of the collimator output measurement, is equivalent to the uncertainty of the spatial resolution of the ruler, confirming the feasibility of obtaining high-precision products. From the data, it was also possible to estimate that the real divergence of the manufactured collimator is 0.799°, which, compared to the expected divergence of the accelerator radiation field for a field of 1.42 cm of 0.813°, represents a discrepancy of less than 1.7%. Finally, the parallel projection measurement of the beam in the central axis of the grid showed a divergence of 0.836°, which is compatible with the previous results.

#### 3.4.2. Radiation Shielding Capacity

**Figure 12:**
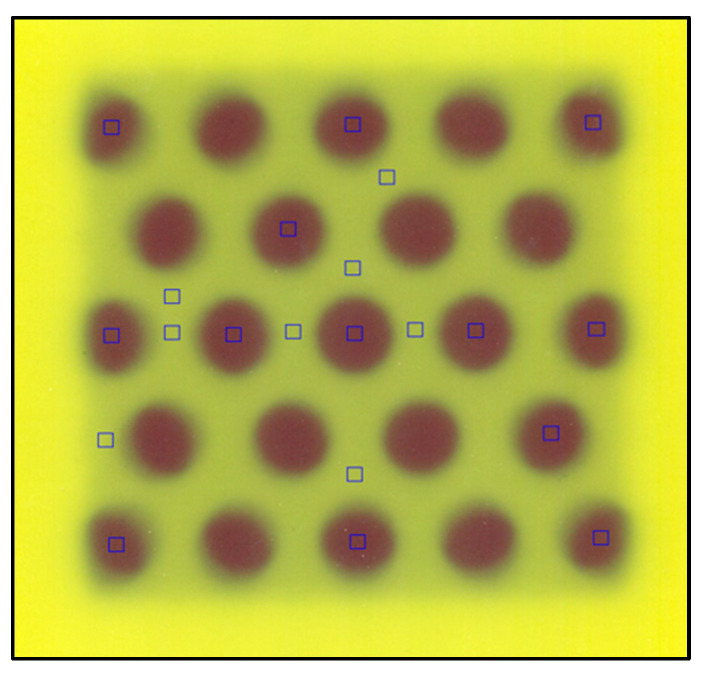
EBT3 radiochromic film irradiated with a 6 MV beam from the UNIQUE linear accelerator, at SSD: 95 cm, field size 10 × 10 cm^2^ and 5 cm depth. Square symbols representing the evaluated ROIs.

Registration of the grid images with a field of 10 × 10 cm^2^ was used to evaluate the transmittance of the radiation from the holes concerning the blocked area in terms of relative dose. Twenty-four points in the image were evaluated, selecting a ROI of 0.076 cm^2^ from the most relevant sectors of the grid: central collimator and collimators of the central hexagon, central inter-collimator blocking zones, as well as collimators and inter-collimator blocking zones in the periphery (Figure 12). 

The evaluation of the dose distribution obtained in the collimator zone (non-blocking regions of the beam) showed values of 85–100% of the dose-normalized to the central collimator, with the lowest dose values in the collimators of the periphery of the grid and an appropriate homogeneity in the collimators of the central hexagon of 98.9 ± 0.9%. A transmittance of 15.6 ± 0.6% was determined for the inter-collimator blocking regions. This percentage transmittance is higher than what could be expected if we consider the value obtained from the first HVL calculated in Section 3.3, where for a thickness of 7.1 cm, a transmittance of only 10.0% should be obtained. The effect of “beam hardening” on poly-energetic beams as they pass through an attenuating medium is well known, where the first HVL (thickness that reduces the transmitted radiation to 50%) can be significantly less than the second HVL (difference between the thickness that reduces transmitted radiation to 25% of its original value, and that which reduces it to 50%), and this may be slightly lower or higher (depending on the energy considered) than the third HVL (difference between the thickness that reduces transmitted radiation to 12.5% of its initial value and that which reduces it to 25%). This occurs because by first filtering the lower energy photons, the effective (average) energy of the radiation beam increases after each HVL (this increase is smaller and smaller for higher order HVLs, or it may decrease for energies greater than 4 MeV). From this actual measurement of the inter-collimator transmittance, it is possible to estimate that the value of the second and third HVL for the studied material would be 3.116 cm and 3.068 cm, which are 45.7% and 43.5% higher than the first HVL, respectively.

**Figure 13:**
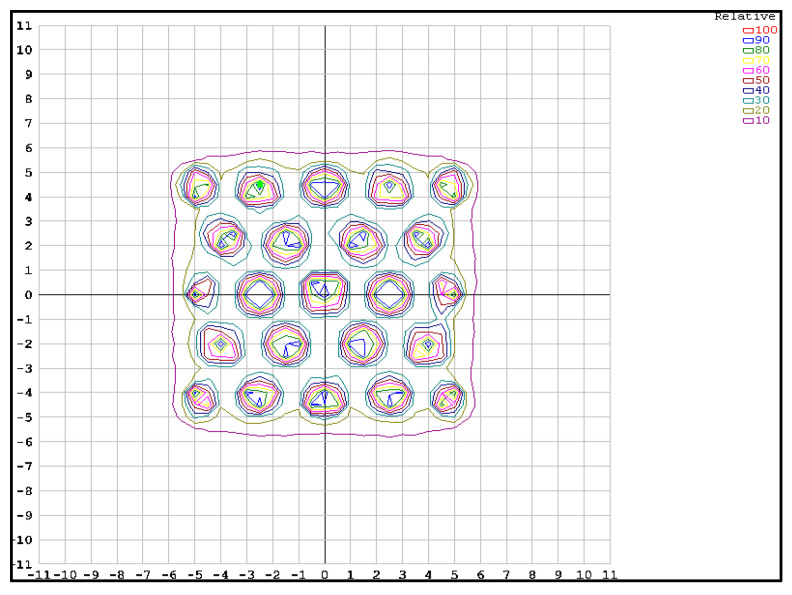
Relative dose distribution of the grid with MapCheck array for a 10 × 10 cm^2^ field size.

Similarly, the relative dose distribution recorded by the isocenter detector array and generated by the collimator grid for a 10 **×** 10 cm^2^ field is shown graphically in Figure 13.

The dose distribution reported by the software in the collimator zone shows an abrupt gradient from 100% of the normalized dose at the center of the collimator to 30% of the dose at a distance of 1 cm from the normalization point. The isodose curve surrounding 50% of the dose in the collimator zone (a criterion used to define the physical size of the collimator in dosimetric terms) showed a diameter of 1.58 ± 0.05 cm diameter. Although this value is 12.8% higher than that measured with the EBT3 film, which in physical terms represents a difference of only 1.8 mm, it must be remembered that this value is obtained by interpolation of the readings of the diodes distributed at a distance of 1.0 cm, merged with an image obtained with the diode array shifted by 0.5 cm to virtually increase the density of the measurement points. Therefore, the discrepancy between the two measurement systems could be explained by the lower spatial resolution of a detector array compared to the micrometer resolution of an EBT3 film. Finally, the inter-collimator zones were shown to be covered by 20% isodose and 10% of the total dose, corresponding to the dosimetric zone known as the umbra, outside the radiation field. Again, we see that the distance between the diodes of the array is not able to register the real dose drop in the inter-collimator block region and overestimates the transmitted dose when interpolating between diodes that are very close to the collimators, making them register part of the penumbra of the collimator radiation field (dose generated outside the physical collimation field since the accelerator emission source is not a point source).

## 4. Discussion

The production of new-generation PLA-W filaments in high concentrations, as reported in this work, offers promising opportunities in the clinical field of radiotherapy. It enables the customized production of various collimators and compensators for highly penetrating radiation, such as that generated by radiotherapy linear accelerators. Moreover, it allows for production in non-specialized spaces (“in situ” in the same clinical center), as it does not require specialized equipment. This offers significant advantages in terms of shortening the logistics chain and availability times of these accessories, reducing costs (by providing an alternative to the few existing commercial options), and enhancing environmental safety by enabling the definitive replacement of highly toxic alloys such as Cerrobend.

The results reported in this work showed the possibility of obtaining pieces with densities up to 7.15 ± 0.01 g/cm^3^ for 100% infill and radiation shielding capabilities that allow a first HVL of 2.138 cm for the 6 MV radiotherapy beam, corresponding to a mass attenuation coefficient of 0.04692 cm^2^/g and an effective energy of the accelerator used of 1.747 MeV. If we compare these values with those obtained by materials traditionally used in the manufacture of collimator blocks, we find that the density of the toxic Cerrobend alloys (9.64 g/cm^3^) [52] is 34.9% higher, allowing for a smaller HVL (1.549 cm) [52]. However, for currently commercialized collimating grids made of bronze, their density is only 18% higher than that obtained with PLA-W in this work [34]. Nonetheless, the higher Z of tungsten allows PLA-W to have a mass attenuation coefficient that is 4.4% higher than that of bronze, which translates into a linear attenuation coefficient that does not differ by more than 14% from the bronze coefficient (0.3352 cm^−1^ and 0. 3922 cm^−1^ for PLA-W and bronze, respectively) [26], allowing the creation of collimating grids with similar physical dimensions to achieve comparable radiation attenuation levels [34].

The study of the effect of different printing parameters on the final result of the manufactured parts showed that the deposition speed has a greater effect on the final density when using layer heights below 50% of the nozzle size, with more consistent densities (<1% variation) being achieved for layer heights of 75% over a wide range of deposition speeds (52.5–105 mm/s). Similarly, spatial congruence showed better results for the 0.45 mm layer height, with physical dimensional discrepancies of less than 0.38% with respect to the design. These parameters are consistent with values reported in the characterization of PLA doped with other metals, such as stainless steel [23,53,54]. Additionally, the nozzle temperature was the variable that showed the most critical variations in the final density of the parts, with temperatures of 205 °C producing densities 16% lower than those obtained at 235 °C. From these values, it can be concluded that maximization of the radiation shielding effect was observed at high deposition rates (105 mm/s), layer heights of 0.45 mm (corresponding to 75% of the nozzle used), and high extrusion temperatures (235 °C).

In terms of precision, the material exhibited spatial congruence between the designed and manufactured parts at sub-millimeter scales, with no measurable changes observed over the course of a year, ensuring stable dosimetry.

Regarding mechanical strength, consistent findings were observed, aligning with previous reports on PLA doped with metal powders. PLA-W demonstrated reductions in Young’s modulus compared to PLA, ranging from 21% to 16%, with the smallest decrease noted for a layer height of 0.45 mm. Tensile stress exhibited dramatic reductions from 89% to 75% for conventional PLA, resulting in abrupt rupture without elongation phase. However, the observed poor mechanical performance values are unlikely to result in deformations due to tensile stresses generated by the weight of the material itself, nor from the expected stresses that the grid would experience when suspended as an accessory at different angular positions, or from the rotational stresses generated by the reduced angular velocity at which the gantry of a clinical accelerator moves.

Another aspect to consider is that most polymeric compounds undergo cross-linking or structural degradation when exposed to radiation [18]. Although effects on the mechanical properties of PLA have been reported, these would not include changes in its structure, even when evaluated at doses of up to 50 kGy (equivalent to approximately 25,000 conventional radiotherapy treatment fractions) [55]. These conclusions suggest that PLA, in conjunction with W, forms high-performance composites in radiation therapy and the stability demonstrated in these studies would allow not only the potential to generate patient-specific grids but also to produce generic grids that can be used by different patients over time, similar to commercially available grids.

## 5. Conclusions

The new generation of high-density PLA-W filaments has provided the first complete characterization of the physical and radiological properties of large parts manufactured by MEX. Among the most relevant qualities, we can mention:

- The achieved densities (7.15 ± 0.01) and the material’s high effective atomic number allow it to attain linear attenuation coefficients at MV photon energies, similar to commercially existing materials manufactured by traditional methods (0.3352 cm^−1^), with radiation shielding capability provided by HVL of 2.138 cm.
- The manufactured pieces exhibited excellent spatial congruence at sub-millimeter resolution and high homogeneity in material distribution and deposition, remaining stable over the course of 1 year.
- The decrease in mechanical properties due to the presence of a high concentration of W granules that break into PLA fiber mesh does not critically affect its stability for the intended application, far from values that could cause structural deformations, while maintaining radiation spatial modulation with great precision.
- The manufactured grid showed levels of dosimetric precision and spatial modulation of radiation through its radiation shielding/attenuation capacity in inter-collimator spaces, with values similar to commercially available grids (transmittance of 15% relative to the central axis) produced by traditional manufacturing methods.

## Figures and Tables

**Table 1:** Manufactured specimens with their respective influence parameters to be evaluated.

Sample	Layer Height (mm)	Deposition Speed (mm/s)	Nozzle Temperature (°C)
1	0.15	52.5	215
2	0.15	70	215
3	0.15	105	215
4	0.30	52.5	215
5	0.30	70	215
6	0.30	105	215
7	0.45	52.5	215
8	0.45	70	215
9	0.45	105	215
10	0.30	70	205
11	0.30	70	225
12	0.30	70	235

**Table 2:** Printing parameters of PLA and PLA-W specimens for evaluation of mechanical properties of PLA-W composite.

Adjustment Parameters and Printing Characteristics	
Extruder nozzle diameter (mm)	0.6
Deposition speed (mm/s)	70
Extruder temperature (°C)	215
Layer height (mm)	0.15, 0.30, 0.45
Infill percentage (%)	100
Diameter of both strands (mm)	1.75

**Table 3:** Concentration in the weight-weight percentage of PLA-W compound.

Material	%*w/w*
H	0.00386
C	0.03450
O	0.03064
W	0.93100

**Table 4:** Physical analysis of the 12 PLA-W slabs with measured physical dimensions, weight, and density was conducted according to ASTM D792 standards [44].

Samples	Length (cm)	Width (cm)	Thickness (cm)	Mass (g)	Density (g/cm^3^)
1	1.940	1.950	0.175	4.8229	7.30
2	1.830	1.830	0.166	4.0136	7.22
3	1.940	1.950	0.173	4.5862	7.03
4	2.000	1.950	0.176	4.8169	7.02
5	1.977	1.978	0.177	4.7807	6.91
6	2.005	2.005	0.174	4.6583	6.66
7	1.993	1.992	0.180	4.8488	6.79
8	1.993	1.991	0.182	4.8345	6.71
9	1.991	1.994	0.176	4.7261	6.78
10	1.880	1.880	0.179	3.8089	6.02
11	2.010	2.005	0.180	4.8486	6.71
12	2.018	2.018	0.168	4.8942	7.18

**Table 5:** Dimensional measurements and weight of cubes.

Infill	Year 2023			Year 2024		
	Length (cm)	Width (cm)	Thickness (cm)	Length (cm)	Width (cm)	Thickness (cm)
15%	0.964±0.002	0.964 ± 0.002	1.006 ± 0.002	0.962 ± 0.002	0.963 ± 0.002	1.006 ± 0.002
50%	0.976 ± 0.002	0.972 ± 0.002	1.006 ± 0.002	0.971 ± 0.002	0.970 ± 0.002	1.004 ± 0.002
100%	1.009 ± 0.002	1.008 ± 0.002	1.008 ± 0.002	1.010 ± 0.002	1.008 ± 0.002	1.009 ± 0.002

**Table 6:** Mechanical analysis of PLA and PLA-W specimens.

Material	Layer Height (mm)	Young Module (MPa)	Energy at Maximum Tensile Stress (J)	Tensile Strength (MPa)
PLA	0.15	2932.04	0.19	52.69
	0.30	2741.82	0.18	50.03
	0.45	2784.30	0.17	51.42
PLA-W	0.15	2316.58	0.02	10.06
	0.30	2293.17	0.02	12.24
	0.45	2347.98	0.02	12.91

**Table 7:** Pixel intensity values [a.u.] of MV images after the 8-bit grayscale calibration process for the 12 samples under study.

Sample	1	2	3	4	5	6	7	8	9	10	11	12
Value	0.109	0.131	0.181	0.147	0.032	0.100	0.145	0.166	0.024	0.076	0.131	0.181
STD	0.053	0.054	0.051	0.061	0.045	0.050	0.054	0.056	0.041	0.045	0.053	0.056

**Table 8:** PLA-W mass attenuation coefficients for a range of 1–6 MeV.

Energy	1 MeV	1.5 MeV	2 MeV	3 MeV	4 MeV	5 MeV	6 MeV
**μ_m_**	6.628 × 10^−2^	5.550 × 10^−2^	4.461 × 10^−2^	4.042 × 10^−2^	3.950 × 10^−2^	3.970 × 10^−2^	4.039 × 10^−2^

**Table 9:** Lineal attenuation coefficient of PLA-W as a function of 6 MV linear accelerator energy from its HVL and mass attenuation coefficient.

Energy [MV]	First HVL [cm]	μ_m_ [cm^2^/g]	Effective Energy Beam [MeV]	Density [g/cm^3^]	μ [cm^−1^]
6	2.138	0.04692	1.747	6.91	0.3242

## Data Availability

Data are contained within the article.
